# Exploring the relevance of the type of horizontal separator to optimize the extraction efficacy for the Arbequina variety

**DOI:** 10.3389/fpls.2024.1395701

**Published:** 2024-06-07

**Authors:** Abdelaziz Boudebouz, Juan-F Hermoso, Esteve Martí, Antonia Ninot, Ricard Boqué, Montserrat Mestres, Laura Aceña, Gianluca Veneziani, Roberto Selvaggini, Maurizio Servili, Agustí Romero

**Affiliations:** ^1^ Department of Analytical Chemistry and Organic Chemistry, Chemometrics and Sensorics for Analytical Solutions Group (ChemoSens), Universitat Rovira i Virgili, Tarragona, Spain; ^2^ Institute of Agrifood Research and Technology (IRTA), Nuts and Olive Growing Group, Constantí, Tarragona, Spain; ^3^ Department of Agricultural, Food and Environmental Sciences, University of Perugia, Perugia, Italy

**Keywords:** oil extractability, milling technology, 2-phases decanter, processing regulations, pomace

## Abstract

The productivity of virgin olive oil depends not only on agronomic factors but also on the technological factors of the extraction process. The ‘Arbequina’ variety has extractability problems, which is a challenge for master millers anywhere. This work aims to evaluate the behavior of different decanters and seeks to modulate the effect of some processing parameters and their interactions with oil extraction efficiency in the case of ‘Arbequina.’ Fruit characteristics, processing parameters, and extractability were collected over 10 years from 38 decanters that belong to five different brands. The results have shown that fruit moisture is the most relevant factor for oil extractability, especially over 52%. Furthermore, extractability is positively correlated with malaxing temperature, addition of water, and total fat content in the fruit. However, the results show that before applying a regulation, the type of decanter must be considered. The model used in this study has allowed us to optimize the regulations for each type of decanter to reduce oil losses within the pomace, achieving an extraction efficiency within the range of 78%–91.5%. In fact, the best extraction efficiency results (91.5%) were obtained by processing at temperatures >26°C and water injection of 5%.

## 1 Introduction

The production of virgin olive oil (VOO) has increased widely in the last decade due to the growing interest in the consumption of this product around the world (IOC, 2020; Boudebouz, 2021; Xia et al., 2022). Its production is exclusively by physical–mechanical procedures or at least with the addition of specific adjuvants, which often leads to difficulties in reaching a sufficiently good extraction efficiency (Caponio et al., 2016; Khaleghi et al., 2023; Tamborrino et al., 2023). Although new processing technologies, such as the application of heat exchangers, ultrasound, pulsed electric field, microwaves, and vacuum technology, can improve oil extraction, it is always important to consider the interaction between the characteristics of the olives and the extraction device (Taticchi et al., 2013; Gila et al., 2015; Pérez et al., 2021; Sánchez et al., 2022; Khaleghi et al., 2023; Nardella et al., 2023).

On the other hand, the olive sector has been in search of varieties with characteristics adapted to the new productive systems and that are easier to handle and that ensure good productivity in quantitative and qualitative terms, reducing the management cost. In this sense, the ‘Arbequina’ variety offers characteristics suitable for super-high density (SHD) orchards. Managed in this way, this olive cultivar can be harvested mechanically, has an average oil yield (17%–20%) and produces oils with a balanced aromatic profile, and is well-valued by consumers (Benito et al., 2012; Centeno et al., 2020). In fact, it is the most widespread variety worldwide in recent years, mainly in the biggest SHD orchards (Benito et al., 2012; García et al., 2016).

Nowadays, VOO is mainly produced by large-capacity olive oil mills that allow a large amount of olives to be processed in a short time, to improve the quality standard of the final product. However, in many cases, virgin olive oil extraction presents some difficulties due to the variability of many factors, such as the cultivar, fruit characteristics, ripening stage, growing area, climatic season, and agronomic practices. It is a fact that processing olives with high moisture causes the so-called “difficult paste,” which is characterized by oil–water emulsions that are difficult to break. Even more, these emulsions can be stabilized by pectic residues within the colloidal phase produced after crushing the olives. Thus, the combination of these problems is a handicap for horizontal centrifuges and can bring about large oil losses through by-products (Beltrán et al., 2003; Tamborrino et al., 2014). These problems are common in the case of ‘Arbequina,’ specifically when olives come from an early harvest of irrigated SHD orchards (Benito et al., 2012; García et al., 2016). The processing of these “difficult pastes” generates significant oil losses, which requires special attention during the preparation of the olive paste and along the oil separation steps. In fact, the right management and control of the different phases of the VOO mechanical extraction process, such as the crushing step and conditioning of olive paste, can have a significant impact on the rheology of the olive paste with a high effect on the oil yield (Perone et al., 2023). The treatment of olive paste before the extraction phase was recently the subject of several studies concerning the introduction of emerging technologies in olive oil mechanical extraction plants such as the application of heat exchangers, ultrasounds, microwaves, and vacuum systems able to modify the physicochemical structure of the olive paste (Puertolas and de Maranon, 2015; Bejaoui et al., 2016; Tamborrino et al., 2021; Taticchi et al., 2019; Veneziani et al., 2022; Juliano et al., 2023).

The processing conditions such as the malaxation time and temperature, the use of technologic adjuvants (water and talc), the horizontal centrifuge force and inner geometry, the diaphragm diameter, the internal deposition point, and the differential speed, among others, are factors with a direct influence on both the extraction efficiency and the quality of the oil obtained (Kalua et al., 2006; Altieri et al., 2013; Esposto et al., 2013; Caponio et al., 2016; Kalogianni et al., 2019; Taticchi et al., 2021).

Choosing the settings for these parameters is the decision of the master miller, which depends on the particular options of the decanter, the fruit characteristics, and the strategy of the company. Among the different options for the master miller, there is a set of regulations that have the greatest impact with the least interference on the process, such as temperature and time of malaxation, paste injection rate and water injection, or the addition of adjuvants (Kalua et al., 2006; Gila et al., 2015; Boudebouz et al., 2021; Hermoso et al., 2021; Leone et al., 2021). However, not all cultivars have the same performance for each of these processing regulations. ‘Arbequina’ presents several difficulties that reduce its industrial extractability, especially when it comes to fruits with a high moisture content.

Regarding the quality characteristics of VOO, the master miller must opt for a suitable balance between these and the oil yield. In fact, some processing conditions can affect the olive oil quality, mainly phenolic and volatile compounds, the main molecules responsible for VOO health and sensory properties. This is the case of the addition of certain amounts of water during malaxation or into the decanter, which, on the one hand, reduces the final amounts of polyphenols in the oil and, on the other hand, improves the oil yield under certain conditions. It is generally accepted, operating in a two-phase system, to use up to 10% of water with respect to the olive paste during the production of extra virgin olive oil with a very low reduction in the final polyphenol content of the oil. In addition, some new instruments are currently being developed (i.e., the IMS injector) to moderate this addition of water into the horizontal centrifuge with minimal interference in the quality of the olive oil (Leone et al., 2021).

The production of virgin olive oil in Catalonia is estimated at approximately 30–40 tons per year and has an olive area that exceeds 110,000 ha, distributed mainly in the southeast of the Catalan territory, with the ‘Arbequina’ being the main variety destined for olive oil production. Its production comes mainly from intensive and SHD orchards with irrigation, which produce fruits with a high moisture content (generally higher than 50%), causing some difficulties during processing (Benito et al., 2012; García et al., 2016). The milling facilities in Catalonia are of medium size with a theoretical production capacity between 500 and 10,000 kg/h and generally operate with the two-phase extraction system (Boudebouz et al., 2021, 2023).

The IRTA has more than 20 years of experience in the processing of ‘Arbequina’ fruits of different characteristics and origins according to a quality improvement program funded by the Catalan Government, with the participation of more than 30 olive mills every year. Thus, there is a huge amount of data that permits figuring out the relationship between ‘Arbequina’ olive characteristics and the performance of different types of milling facilities. The olive milling facilities in Catalonia include a range of different machine builders, and the know-how of the master millers suggests that not all horizontal machines perform equally to a particular setup.

This study aims to assess this differential behavior of different horizontal centrifuges and seeks to modulate the effect of some processing parameters, usually applied in any conventional olive oil mill operating in a two-phase system, to obtain and provide optimal conditions to increase the extraction efficiency of virgin olive oil during the processing of ‘Arbequina.’

## 2 Material and methods

### 2.1 Data collection

The work was carried out based on data collected from different productive areas of Catalonia over a period of 10 years (2011–2021), through scheduled technical visits to each milling facility.

During these technical visits, all processing conditions were verified, and samples of olives and pomace were taken. For each sample, the total fat and moisture contents were measured in order to determine the oil extraction efficiency and to determine which factors most influence oil extractability. Thus, processing parameters adjusted by the master miller, such as sieve size, malaxation time and temperature, addition of talc, paste injection rate, addition of water, diaphragm diameter, and horizontal centrifuge capacity, were taken into consideration.

For each sample, the following terms were considered:

• Humidity (%*_H*) expressed as a percentage of water in the sample. It was measured gravimetrically from approximately 50 g of either grounded olives or olive pomace, after drying in an air oven at 105°C for 24 h (UNE 55020 Standard).

• Total fat content (% dry basis) expressed as a percentage of total fats present in the sample with respect to dry basis. It was measured from the samples previously dried in an air oven at 105°C, and the total fat content was determined by solvent extraction (n-hexane), in a Soxhlet extractor, for a minimum of 6 h (Soxhlet, UNE 55030 Standard).

• Extraction efficiency (Ext_%) expressed as a percentage of the oil extracted. It explains the efficiency of industrial oil extractability and it is reflected as the ratio between the amount of oil extracted and the total fat content available in a determined quantity of olives.

• Paste injection rate (*P-Inject_%*) expressed as a percentage and calculated regarding the quantity of olive paste injected (kg/h) with respect to the total capacity of the horizontal centrifuge. In total, the data considered were collected from 26 different mills representing a variability of machinery from five commercial brands (in order to avoid a direct mention of private brands, they will be called “A,” “B,” “C,” “D,” and “E”) and a theoretical capacity that ranges between 1,000 kg/h and 10,000 kg/h (**Table 1**). Each decanter brand has its own geometric and power characteristics, meaning that differences in oil extractability are expected when fruits of the same ‘Arbequina’ cultivar are processed.

**Table 1:** Distribution of the different brands and capacities of the decanters used in the study.

Brand	N° Mills	N° decanters	Capacity (Kg/h)					Total observations
			< 1000	[1000 - 2999]	[3000 - 4999]	[5000 - 6999]	≥ 7000	
**A**	4	4	–	–	3	1	–	179
**B**	1	2	–	–	2	–	–	42
**C**	1	2	–	–	–	1	1	80
**D**	14	21	1	9	5	2	3	692
**E**	6	9	1	1	5	2		287
**Total**	26	38	2	10	15	6	4	1280

• Water injection rate (*W-Inject_%*) expressed as a percentage and calculated regarding the amount of water (L/h) injected into the horizontal centrifuge with respect to the olive paste injection (kg/h).

Finally, to obtain representative and reliable results, in this study, a data matrix of 1,117 rows (*N*) including a wide range of columns that define fruit characteristics and processing parameters adjusted was used.

### 2.2 Data analysis

The statistical analysis was performed using the Minitab v.21.3 software. The influence of the processing parameters (malaxation time, temperature, paste injection rate, water injection rate, diaphragm diameter, brand, capacity, talc, and sieve size) and their interactions with oil extraction efficiency was analyzed by applying the Pareto chart of standardized effect. The Pareto chart displays a frequency histogram where the length of each bar on the diagram is proportional to the absolute value of its associated standardized effect. Bars that extend beyond the vertical line in the chart indicate which effects are statistically significant at the 95% confidence level. In addition, to find out possible interactions between the different processing parameters, oil extractability, and oil losses within the pomace, a correlation analysis was applied to the data matrix.

Moreover, a CART (classification and regression tree) regression was used to create an optimal decision tree to assess the oil extraction efficiency. CART regression illustrates important patterns and relationships between a continuous response (oil extractability) and important predictors within highly complex data, including predictive continuous variables (i.e., fruit humidity and processing parameters) and categorical variables (i.e., brand machinery). The decision tree allows to identify and separate the variables according to their importance on the oil extraction efficiency. To validate the model, a 10-fold cross-validation was applied to the CART^®^ regression.

## 3 Results and discussion

The extraction efficiency (extractability) explains the recovery of oil in comparison with its total amount in the olives. It is inversely related to the fat content in the pomace and depends on different factors such as the characteristics of the fruit, the preparation of the olive paste (milling, malaxation), and the centrifugation conditions (Gila et al., 2015; Khaleghi et al., 2023). Thus, the extractability was correlated with the different processing factors, in order to know which of them had the greatest relative influence on the oil extraction. The results obtained in this study explain a great variability in the extraction efficiency of ‘Arbequina,’ since the data were obtained directly from oil mills that operate under different processing conditions and have different brand machinery.

The results of pomace fat content showed average values ranging from 9% to 14% on a dry basis (db), reaching an extraction efficiency within the range of 78%–91%, in agreement with the results of oil extractability reported in other studies for the ‘Arbequina’ cultivar (Boudebouz et al., 2021; Hermoso et al., 2021; Khaleghi et al., 2023; Perone et al., 2023). It must be pointed out that, during virgin olive oil production, oil losses of up to 10% through pomace are traditionally accepted for this variety.

**Figure 1:**
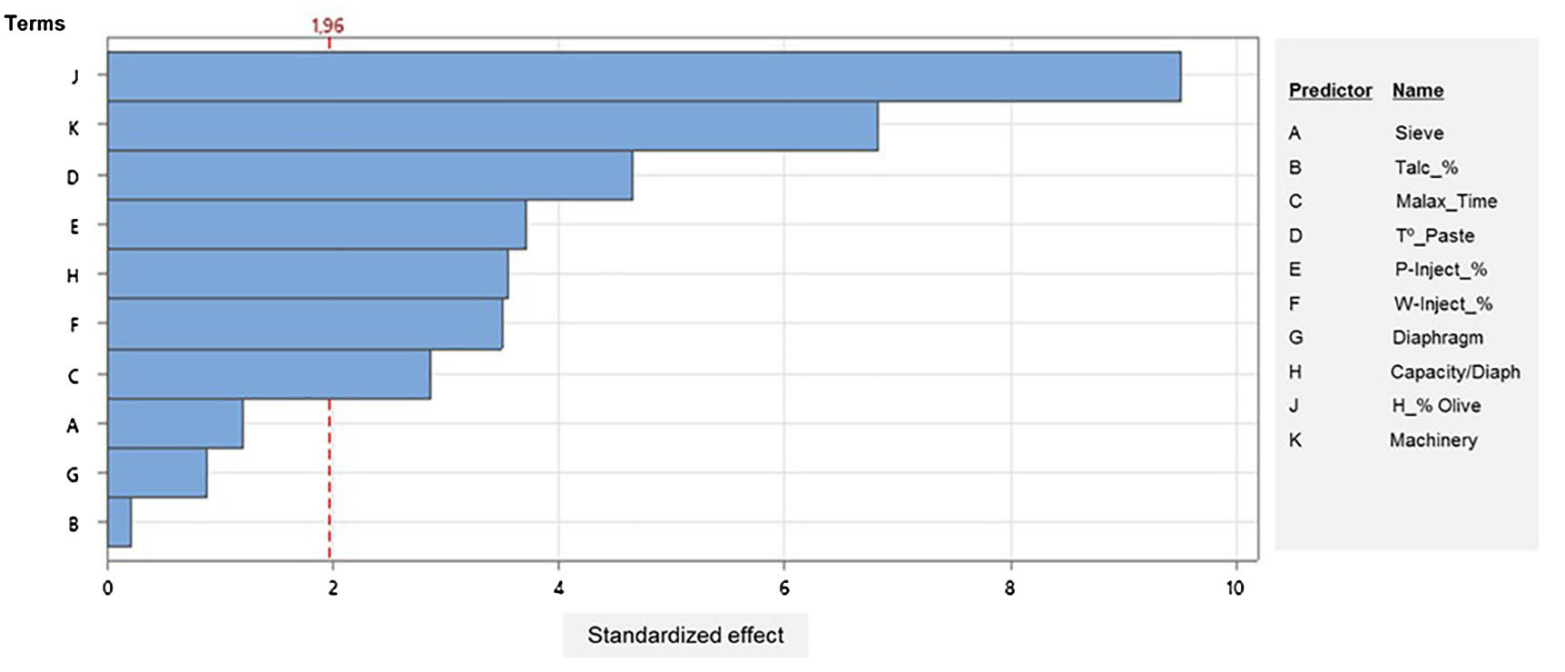
Pareto chart of the standardized effect of different parameters on the oil losses within the pomace (*α* = 0.05).

To score the relative effect of the different factors on fat loss through pomace, a Pareto chart of standardized effects was performed on the data matrix (**Figure 1**). The Pareto chart shows the absolute values of the standardized effects from the largest effect to the smallest effect. The results showed that the greatest variability depends on the fruit moisture content, in agreement with previous studies that relate the oil yield with fruit characteristics, such as ripening, pectic substances, and moisture and oil content (Kalogianni et al., 2019; Perone et al., 2023). In addition, it can also be seen in the Pareto chart that the machinery, characterized by specific technological characteristics, and malaxation temperature are factors of great relative impact that must be taken into account when setting up the processing conditions (Altieri et al., 2013; Esposto et al., 2013).

Furthermore, the adjustments of the injection parameters of the decanter (injection of paste and water) also showed a significant effect but with less relative importance. Nevertheless, it is essential to indicate that a correct application of these adjustments depends on the functional and geometric characteristics of the decanter, such as its capacity, the diameter of the diaphragm, and the differential and centrifugal force, as well as the characteristics of the fruit and the preparation conditions of the paste (Iqdiam et al., 2018; Boudebouz et al., 2021; Leone et al., 2021). Finally, it should be noted that the sieve size, the addition of talc, and the diaphragm diameter are regulations used only by a few master millers in Catalonia and even their use is more qualitative than quantitative. In fact, master millers who use them decide between two levels of regulation (low and high). This could explain the very low level of impact of these factors though they are widely accepted as very useful from a theoretical point of view.

**Figure 2:**
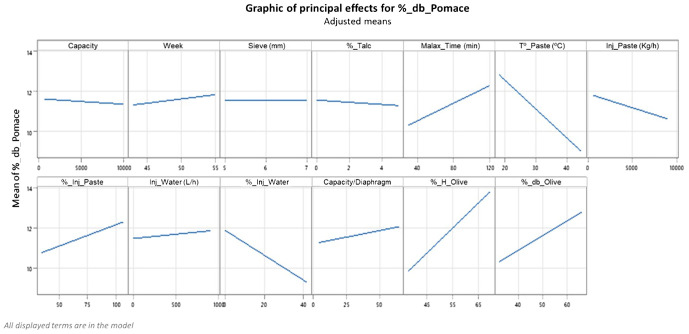
Principal effect of each parameter on the oil losses within the pomace.

Regarding the main effects of the different processing parameters in the model, **Figure 2** shows the individual influence of each parameter on the variability of the fat content in the pomace (oil losses). The results indicate that the different processing parameters considered in the model may have an independent response to the oil losses within the pomace.

In fact, the regression model used in this study shows that the greatest variability is caused by fruit moisture and paste temperature, followed by water-to-paste ratio and fruit oil content. The oil losses increase as fruit moisture increases, with an average increase of almost four points in the losses (from <10% db to 14% db, respectively) when fruit moisture increases from 40% to 67%. An explanation for this behavior could be the fact that the high moisture content generates the so-called “difficult paste,” causing emulsions and instability of the paste’s viscosity, making it difficult to handle (Tamborrino et al., 2014). A reduction of these extraction problems can be obtained with careful management of the olive paste preparation steps (crushing and malaxation) and the right setting of technological parameters of the separation phase (Squeo et al., 2017; Caponio et al., 2018; Vallone et al., 2022; Nardella et al., 2023; Perone et al., 2023).

Regarding the processing factors, the increase in temperature facilitates oil extraction. As can be observed in **Figure 2**, the losses decrease from 13% db to 9% db when the processing temperature rises from 20°C to 42°C. However, this option does not seem to be an adequate solution to improve the oil extractability for ‘Arbequina,’ since the increase in temperature negatively affects the quality of the oil, especially beyond 35°C (Kalua et al., 2006; Kalogianni et al., 2019). In fact, high processing temperatures are able to determine a significant reduction of the activity of the lipoxygenase pathway (LOX) and an increase of the oxidation phenomenon due to polyphenoloxidase (PPO) and peroxidase (POD) with a consequent negative impact on the concentration of volatile and phenolic compounds (Luaces et al., 2007; Taticchi et al., 2013; Marx et al., 2021).

Unexpectedly, the malaxation time shows an increase in oil losses through the pomace, increasing on average from 10% db to 12% db when the time is increased from 30 to 120 min. This could be explained by the fact that oil losses are more affected by malaxation time when the moisture of the olives is below 45%, whereas above this moisture level, a longer malaxation time does not lead to higher oil loss. In fact, below 45% moisture, the paste in the malaxer could become difficult to handle due to excessive evaporation of water during the first minutes of malaxation; water loss in higher moisture pastes might not be as critical and the final consequence is that on average there seems to be a direct relationship between malaxation time and oil losses.

On the other hand, the addition of water in terms of quantity (W-inject L/h) does not seem to have a clear effect on oil losses within the pomace, but a moderate addition of water with respect to the amount of paste injected (%_Water/Paste) from 10% to 40% results in a reduction of oil losses from 12% db to 9% db, possibly because it helps in the formation of the rings and the separation of different phases inside the decanter, so it can be an effective option to improve the extractability in ‘Arbequina.’ However, this parameter must be applied with great care since it directly influences the quality of the oil obtained, especially the phenolic profile (Altieri et al., 2013; Leone et al., 2021).

Concerning the increase in P-Inject (kg/h), it seems to decrease oil losses within the pomace but in a very low proportion, whereas the injection of larger amounts of olive paste compared to the total capacity of the decanter (P-Inject_%) reduces clearly its residence time inside the drum of the horizontal centrifuge, which results in less oil separation (Altieri et al., 2013; Boudebouz et al., 2021).

According to the regression model used in this study, the adjustment of the diaphragm, the addition of talc, and the size of the sieve do not seem to have a significant effect on oil losses although other studies reported certain effectivity (Caponio et al., 2016). As has already been mentioned, this behavior could be due to the fact that the experimental design in this research does not allow us to study the individual effect of these parameters because many master millers in Catalonia use them following a dichotomic way (small/high diaphragm, yes/no talc, small/high sieve).

**Figure 3:**
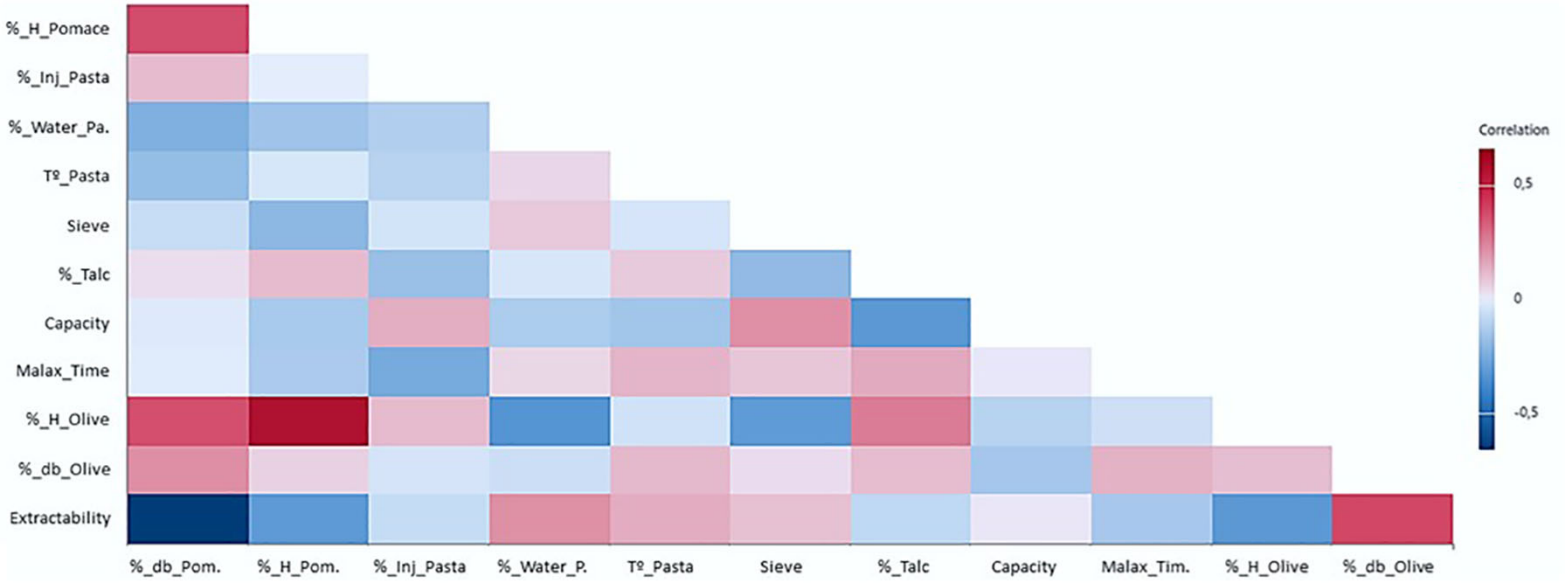
Correlations between the different processing parameters considered in the analytical model.

For the setting of different processing parameters, which basically depend on the industrial strategies of the mills, all processing steps must be considered. In other words, to change a parameter, it is necessary to know the relative effect that this parameter has on the extraction process in comparison to other parameters (i.e., before changing a parameter, it will be recommended to verify if it is possible to act on other parameters with higher relative impact, especially when changes require machinery to stop or seriously affect quality). Moreover, the correlation analysis performed on all the variables considered in the model (**Figure 3**) shows the presence of some interactions between the different processing parameters. Thus, the oil losses within the pomace (% db_pomace) negatively correlate with % H_Olive and % H_Pomace. However, the extractability positively correlates with the total fat content in olives (% db_Olive), water-to-paste ratio (%_Water/Paste), and paste temperature (T°_Pasta).

**Figure 4:**
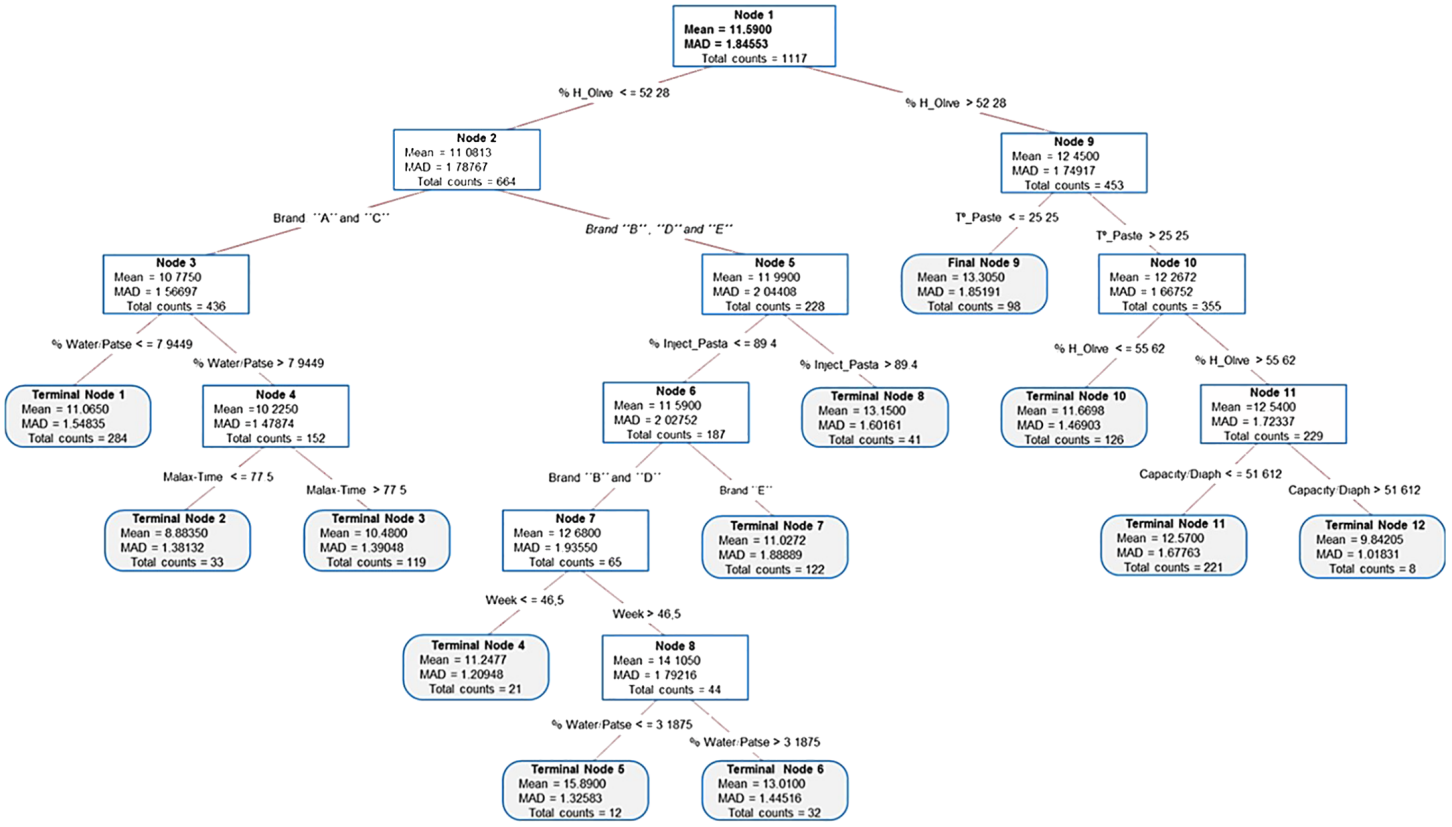
Optimal tree diagram for the predictive effects of different processing parameters, including the machine type, on the oil losses explained in percentage with respect to dry matter.

To consider the best setting options, an optimal decision tree (**Figure 4**) was created using CART on the data matrix. The analytical model selects the best-performing splits based on the effectiveness of the different processing parameters (predictors) on the variability of the total fat content in pomace (response) to create an optimal tree that leads to terminal nodes with the best results for the oil losses within the pomace. In each classification (node), the predictor with the best effectiveness, the number of cases counted (data rows), the mean of fat content in the pomace, and the mean absolute deviation (MAD) are represented in the tree layouts. This analysis includes the machine type as a predictor.

### 3.1 Optimal regression tree for the total fat losses within the pomace

According to the regression model (shown in **Figure 4**), the main variability in total pomace fat content is due to % H_Olive. Processing olives with moisture higher than 52% generates greater losses with a mean value of 12.45% db (*n* = 453). In this case, increasing the temperature above 25°C can be an effective solution to improve oil recovery, with an improvement of more than 1.6% db when the fruit humidity oscillates between 52% and 55% versus a lower temperature. However, when the fruit humidity is higher than 55%, the predictive regression model recommends (in addition to increasing the temperature) the readjustment of the diaphragm according to the decanter characteristics. The higher diaphragm/decanter capacity ratio explains a higher surface of the separating rings inside the decanter. Thus, the model shows a significant reduction of oil losses in the pomace (from 12.45% to 9.84% db) with respect to the higher diaphragm diameter/decanter capacity ratio.

The CART regression model shows better results of oil recovery when fruit moisture is lower than 52%, with a mean value of pomace oil losses of 11.08% (*n* = 664). In this case, the choice of the optimal processing parameters depends on the brand of the horizontal centrifuge, which is basically related to the functional characteristics of each decanter. Thus, the regression model could separate two main groups with a difference of approximately 1.2% db of the total fat losses. This behavior could be due not only to the brand machinery itself but also to the functional characteristics (geometrics and differential), the total capacity, and the fruit characteristics.

In the case of brands “A” and “C,” the oil recovery was improved by the injection of a moderate amount of water (approximately 8% with respect to the paste injected). In this case, oil recovery was not improved by extending the malaxation time; in fact, the model shows better oil depletions with a malaxation time of less than 77 min. Even more, it was observed that a malaxation time of less than 70 min can result in better oil recovery, with a difference of 2.0% db (*n* = 33) compared to a malaxation time of more than 70 min (*n* = 119). However, such a long process should be avoided during the processing of the ‘Arbequina’ variety, especially when it comes to high-quality olive oil production or the processing of larger volumes of olives (Angerosa et al., 2001; Clodoveo, 2012). Regardless of the genetic origin of the olive fruits, a limited time of malaxation process is a practice that must be carried out whenever the master miller wants to extract a VOO of high quality (Kalogianni et al., 2019; Nardella et al., 2023).

In the case of brands “B,” “D,” and “E,” the management of the paste injection rate could be the priority factor to improve oil depletion. In fact, the model shows that processing below 89% of the decanter capacity results in better oil depletion. In addition, an increment of oil losses is observed from week 47 (from 15th November), which could be reduced by the addition of moderate amounts of water (>3%). This behavior could be due to the physicochemical characteristics of the olive paste (i.e., homogeneity, viscosity, and pectic substances), which suggests reducing the paste injection rate and the addition of a moderate amount of water. In fact, the best oil depletion was obtained when operating at 60%–80% of the decanter capacity together with the addition of approximately 8% of water. Operating at a higher injection rate, the olive paste circulates faster inside the decanter drum, which results in less time for the separation of the oil, but the addition of a certain amount of water helps in the separation of the different ring phases (since the water gets between the oil and the solid particles with greater density) (Altieri et al., 2013; Tamborrino et al., 2015). Furthermore, although oil extractability could depend on the decanter brand and its functional characteristics, this is not a conclusive result in this study because these variables were not taken into account previously. Thus, an ANOVA test was carried out to assess the effect of the brand machinery (five brands used in this study) on the variability of oil losses, and the results showed no significant differences. Thus, the variability could be due to the decanter’s functional characteristics and not to the machinery brand.

### 3.2 Optimal regression tree for the extraction efficiency

The average value for the extraction efficiency was 86.7% ( ± 5, *n* = 897), which corresponds to 11.5% db of the total pomace fat losses. The main factors that affected the extraction efficiency were fruit moisture and its total fat content. The processing of fruits with higher fat content increases the amounts of oil extracted and therefore the industrial yield, since oil losses in pomace oscillate in a range of 11.5% db ( ± 2) regardless of the total amount of oil in the olives. However, the processing of olives with moisture higher than 54% reduces the extraction efficiency, as the fruit moisture is a critical factor for oil depletion in ‘Arbequina.’ The processing of olives with such a level of humidity has shown an extraction efficiency of 85.2% on average (*n* = 272), although this value can be lower depending on other factors. In addition, processing at temperatures below 26°C has allowed us to obtain an extraction efficiency of 86.9% (*n* = 241) versus 88.9% (*n* = 308) when it is higher than 26 degrees. Thus, for reducing oil losses, operating at a temperature >26°C together with the injection of a moderate amount of water (approximately 5%) has shown an improvement in extraction efficiency of about an average of 91.5% (*n* = 32).

According to the regression model, the interest in improving the oil quality (by processing at lower temperatures), the correct choice of the diaphragm diameter, and the addition of a moderate amount of water (approximately 4%) is an adequate option for oil recovery, achieving an extraction efficiency of up to 90.2% (*n* = 23).

Finally, as observed for the oil losses within the pomace, prolonging the malaxation time did not show an improvement in extraction efficiency although a slight increase was observed when operating at temperatures above 27°C. This behavior is due to the greater effect of the temperature; however, the quality will be compromised under these conditions (high temperature and long malaxation time can bring about an increase in peroxides). In addition, both high temperature and long time of malaxation have a negative effect on the adequate control of endogenous enzymes involved in the regulation of the final content of VOO phenolic and volatile compounds (Taticchi et al., 2013; Kalogianni et al., 2019; Nardella et al., 2023).

### 3.3 Validation of the regression model for oil extractability

**Figure 5:**
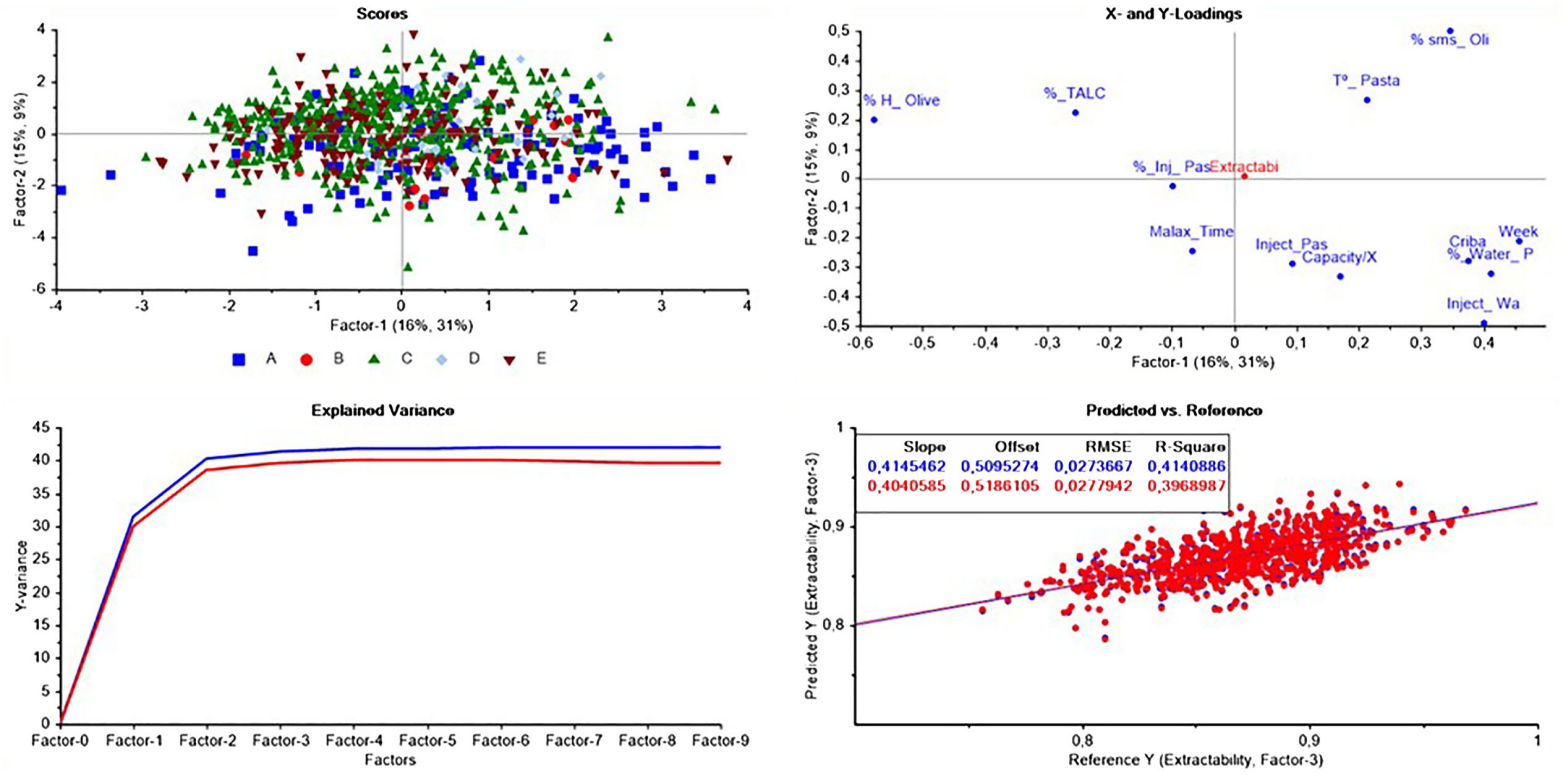
Results of the PLS analysis for the oil extractability as a function of the different processing parameters.

To assess the relationship between the different processing parameters and oil extractability, a partial least squares (PLS) regression model was built using the processing parameters as the *X* variables and the oil extraction efficiency as the *Y* variable (**Figure 5**). As can be seen in the scores plot, the different horizontal centrifuge brands do not show any specific tendency or grouping, which explains that the greatest variability in oil extractability comes from the processing conditions and not from the machinery brand.

The PLS analysis has resulted in a coefficient of determination (*R* ^2^) of 0.414 for calibration and 0.396 for validation with a root mean squared error (RMSE) of 0.027 for both. The low *R* ^2^ can be due to the independent effect of different processing factors, included in the PLS model, on the oil extraction efficiency and the total oil losses within the pomace. Moreover, when looking at the loadings plot, as expected, oil extractability negatively correlates with %_H-Olive (−0.58) and positively correlates with paste temperature (+0.21), total oil content in olives (+0.35), and amount of water injected (+0.21) with respect to factor-1.

## 4 Conclusions

The results of this study showed that fruit moisture is a critical factor for the industrial extractability of virgin olive oil from the ‘Arbequina’ variety. Oil losses in the by-products increase when the processed olives have a moisture content above 52%; however, a good adjustment of the different extraction factors, adequately combined with the right preparation of olive paste controlling the main technological parameters, can reduce these industrial losses.

Furthermore, the relevance of taking into account the horizontal separator characteristics (type, capacity, diaphragm) in order to choose the best combination of processing regulations that permits high extractability has been demonstrated.

The main regulations during the processing of ‘Arbequina’ olives can be predicted by modeling the different processing factors according to their main effect on oil extractability. This may help in the decision to apply correct regulations in order to optimize the extraction efficiency and provide solutions to reduce oil losses. Finally, the modeling of these parameters can be an interesting tool in the sector of olive oil production by applying smart technology to auto-control and optimize the extraction conditions that allow an improvement of the oil extraction efficiency, depending on the fruit characteristics. Furthermore, the modeling of this type of data can be an option of high interest to reduce production costs by introducing new self-control and automation systems for olive oil mills. The large amount of data of this modeling should and could be enriched and also improved with other possible parameters linked to other aspects of the mechanical extraction process to progressively update useful additional information for the increase of VOO extractability.

## Data availability statement

The raw data supporting the conclusions of this article will be made available by the authors, without undue reservation.

## Author contributions

AB: Conceptualization, Data curation, Formal analysis, Investigation, Methodology, Software, Validation, Writing – original draft, Writing – review & editing. JH: Conceptualization, Funding acquisition, Investigation, Visualization, Writing – review & editing. EM: Conceptualization, Investigation, Methodology, Writing – review & editing. AN: Investigation, Project administration, Visualization, Writing – review & editing. RB: Conceptualization, Formal analysis, Methodology, Software, Validation, Visualization, Writing – review & editing. MM: Conceptualization, Investigation, Supervision, Visualization, Writing – review & editing. LA: Investigation, Methodology, Writing – review & editing. GV: Conceptualization, Methodology, Validation, Visualization, Writing – review & editing. RS: Conceptualization, Data curation, Investigation, Writing – review & editing. MS: Conceptualization, Investigation, Supervision, Visualization, Writing – review & editing. AR: Formal analysis, Investigation, Methodology, Supervision, Validation, Visualization, Writing – review & editing.
